# Transcriptional repression of DNA repair genes is a hallmark and a cause of cellular senescence

**DOI:** 10.1038/s41419-018-0300-z

**Published:** 2018-02-15

**Authors:** Guillaume Collin, Anda Huna, Marine Warnier, Jean-Michel Flaman, David Bernard

**Affiliations:** Centre de Recherche en Cancérologie de Lyon, Inserm U1052, CNRS UMR 5286, Université de Lyon, Centre Léon Bérard, 69373 Lyon, France

## Abstract

Cellular senescence response is (i) activated by numerous stresses, (ii) is characterized by a stable proliferation arrest, and (iii) by a set of specific features. Timely regulated senescence is thought to be beneficial, whereas chronic senescence such as during normal or premature aging is deleterious as it favors most, if not all, age-related diseases. In this study, using in-house or publicly available microarray analyses of transcriptomes of senescent cells, as well as analyses of the level of expression of several DNA repair genes by RT-qPCR and immunoblot, we show that repression of DNA repair gene expression is associated with cellular senescence. This repression is mediated by the RB/E2F pathway and it may play a causal role in senescence induction, as single DNA repair gene repression by siRNA induced features of premature senescence. Importantly, activating RB independently of direct DNA damage also results in repression of DNA repair genes and in the subsequent induction of DNA damage and senescence. The dogma is that DNA damage observed during cellular senescence is directly provoked by DNA lesions following genotoxic attack (UV, IR, and ROS) or by induction of replicative stress upon oncogenic activation. Our in vitro results support a largely unsuspected contribution of the loss of DNA repair gene expression in the induction and the accumulation of the DNA damage observed in most, if not all, kinds of cellular senescence, and thus in the induction of cellular senescence. Further demonstration using in vivo models will help to generalize our findings.

## Introduction

The state of cellular senescence is characterized by a stable proliferation arrest and the acquisition of specific features such as morphological, metabolic and transcriptional changes. Timely-regulated senescence is thought to be beneficial as it exerts tumor suppressive activity both by blocking proliferation and by activating immune cells^1–3^, as it contributes to wound healing or as it favors insulin secretion and delays Type I diabetes^4,5^. By contrast, chronic senescence such as during normal or premature aging is deleterious as it favors the development of age-related diseases including cancer. In the context of aging, restricted proliferation of senescent cells limits organ renewal capacities, and the senescent secretome alters the architecture and functions of tissues, both of which are thought to contribute to age-related pathologies including cancer^6–12^.

Cellular senescence can be activated by numerous cellular stresses such as replicative exhaustion, radiation, genotoxicity, oncogenic signals, as well as oxidative stress. They induce senescence, at least in part, through induction of DNA damage and DNA damage signaling. Increased DNA damage observed in senescent cells is thought to be due to physical attacks of the DNA, such as by reactive oxygen species (ROS) or ionizing radiation (IR), and/or to replicative stress after oncogene activation^13–16^.

In this study, we revisited this concept by showing that the repression of DNA repair genes is observed in senescent cells, and that this repression is sufficient per se to result in increased DNA damage and features of cellular senescence induction through an amplifying loop involving P53 and RB factors. Thus, repression of a DNA repair gene program could be a critical step in senescence induction.

## Results

### Senescent cells display decreased expression of DNA repair genes

**Fig. 1:**
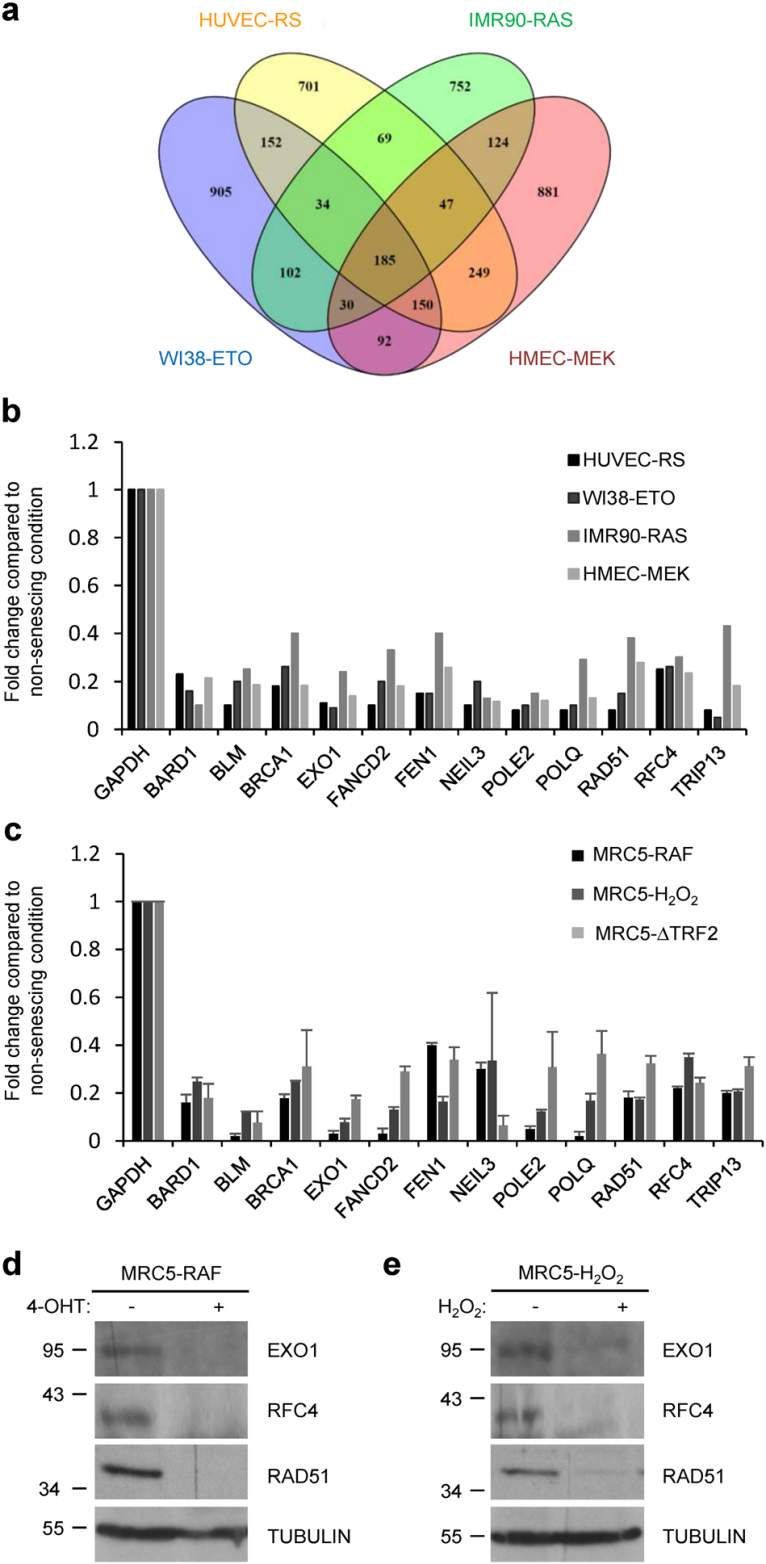
Senescent cells display decreased DNA repair gene expression. **a** Venn diagram analysis showing commonly downregulated genes between 4 gene expression profile datasets: HMEC-MEK (OIS), IMR90-RAS (OIS), HUVEC-RS (replicative senescence), WI30-ETO (genotoxic stress-induced senescence). **b** Gene expression data for 12 DNA repair genes derived from gene profiling datasets described in (**a**). **c** Senescence was induced in MRC5 by three different inducers: RAF overexpression (96 h after 4-OHT treatment), H_2_O_2_ treatment (24 h after 1 h exposure to H_2_O_2_) or a ΔTRF2 expression (10 days after infection). RNAs were prepared and reverse transcribed. Levels of indicated DNA repair gene transcripts were quantified by quantitative PCR and the results were normalized against the level of GAPDH. Means ± SD are presented in the graph. A statistically significant downregulation was observed for all the genes described (*t*-test *P*-value <0.001). **d**, **e** Western blot analyses were performed 96 h after 4-OHT treatment for MRC5-RAF (**d**) and 48 h after 1 h exposure to H_2_O_2_ in MRC5 (**e**) with antibodies targeting the indicated proteins. Tubulin was used as loading control. The different experiments shown are representative of at least two repeats

To gain some insight into the mechanisms regulating cellular senescence in epithelial cells, which are at the origin of most cancers, we characterized the transcriptome of immortalized human mammary epithelial cells expressing a fused inducible (by 4-OHT) MEK:ER oncogene (HMEC-MEK), a model of oncogene-induced senescence (OIS) that we have described previously^17–19^. Gene ontology (GO) analyses revealed that expression of numerous genes involved in pathways covering different DNA repair systems were strongly downregulated during OIS in HMEC-MEK cells (Table 1). To verify whether this downregulation of DNA repair gene is specific to either this cell type or to the senescence inducers we examined other publicly available transcriptomic datasets: replicative senescence in human umbilical vein endothelial cells (HUVEC) (HUVEC-RS)^20^, genotoxic stress (etoposide)-induced senescence in immortalized human fibroblasts (WI38-ETO)^21^, and RAS-induced senescence in human fibroblasts (IMR90-RAS)^22^. A Venn diagram analysis between those four sets of expression data led us to identify 185 common downregulated genes (Fig. 1a). GO analysis of those common repressed genes once again revealed highly significant enrichment (13-fold enrichment; *P*-value <10^−19^) in genes involved in DNA repair pathways (Table 2).

**Table 1:** Gene ontology analysis reveals downregulation of numerous DNA repair genes

GO accession number	GO term	Corrected *P*-value	Count selection	Count total	% Count selection	% Count total	Fold enrichment
GO:0006297	Nucleotide-excision repair (NER)	6.4E−07	13	20	0.81	0.11	7.1
GO:0006298	Mismatch repair (MMR)	4.7E−07	16	30	0.99	0.17	5.9
GO:0006284	Base-excision repair (BER)	9.7E−08	20	43	1.24	0.24	5.1
GO:0000724	Double-strand break repair via homologous recombination (RH)	1.8E−06	24	69	1.49	0.39	3.8
GO:0000725	Recombinational repair	2.5E−06	24	70	1.49	0.39	3.8
GO:0006302	Double-strand break repair (DSB)	2.4E−09	39	126	2.42	0.71	3.4
GO:0009411	Response to UV	1.3E−05	30	110	1.86	0.62	3.0
GO:0006281	DNA repair	2E−23	111	410	6.88	2.31	3.0
GO:0006974	Response to DNA damage stimulus	2.2E−19	141	660	8.74	3.72	2.3

Immortalized human mammary epithelial cells expressing a fused inducible MEK:ER oncogene (HMEC-MEK) was induced to senesce by adding 4-OHT. Microarray analyses (Agilent technology) were performed in control and senescent cells and Gene ontology (GO) analysis has been performed using Genespring software (Agilent technology) on the downregulated genes. Corrected *P*-value: corresponds to Benjamini Yekutelli correction, Count selection: corresponds to the number of GO term genes found downregulated in senescent cells, count total corresponds to the total number of genes belonging to the indicated GO, % Count selection means the number of gene repressed in the senescent cells in the indicated GO reported to the total number of repressed genes in the senescent cells, % Count total: the number of gene in the indicated GO term, and Fold enrichment: the ratio between % count selection and % count total

**Table 2:** DNA repair genes downregulated in common in four different microarray datasets of senescence

Gene symbol	Protein name	WI38 ETO	IMR90 RAS	HUVEC RS	HMEC MEK
BARD1	BRCA1-associated RING domain protein 1	6	3	4.4	4.7
BLM	Bloom syndrome protein	4.8	3.9	9.4	5.5
BRCA1	Breast cancer type 1 susceptibility protein	3.8	2.2	5.6	5.5
CDC45	Cell division control protein 45 homolog	9.5	2.6	10	5.9
CDC7	Cell division cycle 7-related protein kinase	6.5	2	2.7	5.5
CDCA5	Sororin	9.8	3.2	8.5	5.9
CDK1	Cyclin-dependent kinase 1	12.1	2.8	11	7.7
CHAF1A	Chromatin assembly factor 1 subunit A	5.6	2.1	4.6	5.0
CHAF1B	Chromatin assembly factor 1 subunit B	6.2	2	3.7	4.3
DNA2	DNA replication ATP-dependent helicase/nuclease DNA2	3	2.1	4.4	2.8
EXO1	Exonuclease 1	11.3	4.2	9	7.0
FANCB	Fanconi anemia group B protein	3.2	2.3	4.7	3.4
FANCD2	Fanconi anemia group D2 protein	4.8	5.1	8	5.6
FANCG	Fanconi anemia group G protein	5.4	2.2	3	2.6
FANCI	Fanconi anemia group I protein	6.3	3.9	4.7	4.1
FEN1	Flap endonuclease 1	6.7	2.1	6.9	3.8
FOXM1	Forkhead box protein M1	18.4	5.6	7.5	4.5
HMGB1	High mobility group protein B1	2.6	2.1	2.7	2.4
HMGB2	High mobility group protein B2	3.4	9.1	5.1	4.5
KIAA0101	PCNA clamp associated factor	11.3	4.2	4.2	6.4
NEIL3	Endonuclease 8-like 3	4.9	7.5	12.6	9.1
NUDT1	7,8-dihydro-8-oxoguanine triphosphatase	5.8	2.3	2.1	2.4
PARPBP	PCNA-interacting partner	7.6	2.1	3.4	6.8
PIF1	ATP-dependent DNA helicase PIF1	2.0	2.1	8.1	5.4
POLA1	DNA polymerase alpha catalytic subunit	6.5	4	4	2.3
POLE2	DNA polymerase epsilon subunit 2	9.6	6.3	11.5	8.5
POLQ	DNA polymerase theta	10.1	3.4	11.2	7.5
PTTG1	Securin	11.1	4.4	5.5	4.3
RAD51	DNA repair protein RAD51 homolog 1	6.3	2.7	10.7	4.7
RAD51AP1	RAD51-associated protein 1	6.5	5.9	9.7	10.0
RAD54B	DNA repair and recombination protein RAD54B	3.6	2.1	2.1	2.7
RAD54L	DNA repair and recombination protein RAD54-like	7.2	2.1	9.3	3.8
RFC3	Replication factor C subunit 3	4.9	4.8	6.1	9.0
RFC4	Replication factor C subunit 4	3.7	3.2	4.1	4.3
RFC5	Replication factor C subunit 5	5.8	2.5	4.5	4.1
RNASEH2A	Ribonuclease H2 subunit A	9.1	3.4	2.7	2.7
TRIP13	Pachytene checkpoint protein 2 homolog	19.3	2.3	11.3	6.6
UBE2T	Ubiquitin-conjugating enzyme E2 T	6.4	2.4	5.7	4.0
USP1	Ubiquitin carboxyl-terminal hydrolase 1	7.3	5.3	3.7	2.5

Numbers indicate the average fold repression between senescent cells and controls for 39 DNA repair genes observed in WI-38 cells exposed to etoposide (WI38-ETO, GSE62701), in IMR90 cells overexpressing RAS (IMR90-RAS, GSE60652), in HUVEC cells during replicative senescence (HUVEC-RS, GSE7091), and in HMEC cells overexpressing MEK (HMEC-MEK, this study)

To perform further functional analysis, 12 genes, involved in several DNA repair pathways, commonly and strongly down-regulated in the 4 different “senescence transcriptomes” were selected (Fig. 1b). Negative regulation of these 12 DNA repair genes was further confirmed by RT-qPCR in three different models of senescence, in MRC5 normal human fibroblasts (i) overexpressing a fused inducible (by 4-OHT) oncogene RAF:ER (MRC5-RAF) (Supplementary Figure S1), (ii) exposed to H_2_O_2_ (MRC5-H_2_O_2_) (Supplementary Figure S2) (iii) expressing a non-functional TRF2 leading to telomere dysfunction (MRC5-ΔTRF2) (Supplementary Figure S3) (Fig. 1c). Finally, we confirmed that this decrease at the mRNA level resulted in a decrease at the protein level (Fig. 1d, e).

Altogether, these data suggest that a decreased expression of DNA repair genes is a hallmark of cellular senescence.

### Repression of DNA repair genes in senescent cells is mediated by the RB pathway

**Fig. 2:**
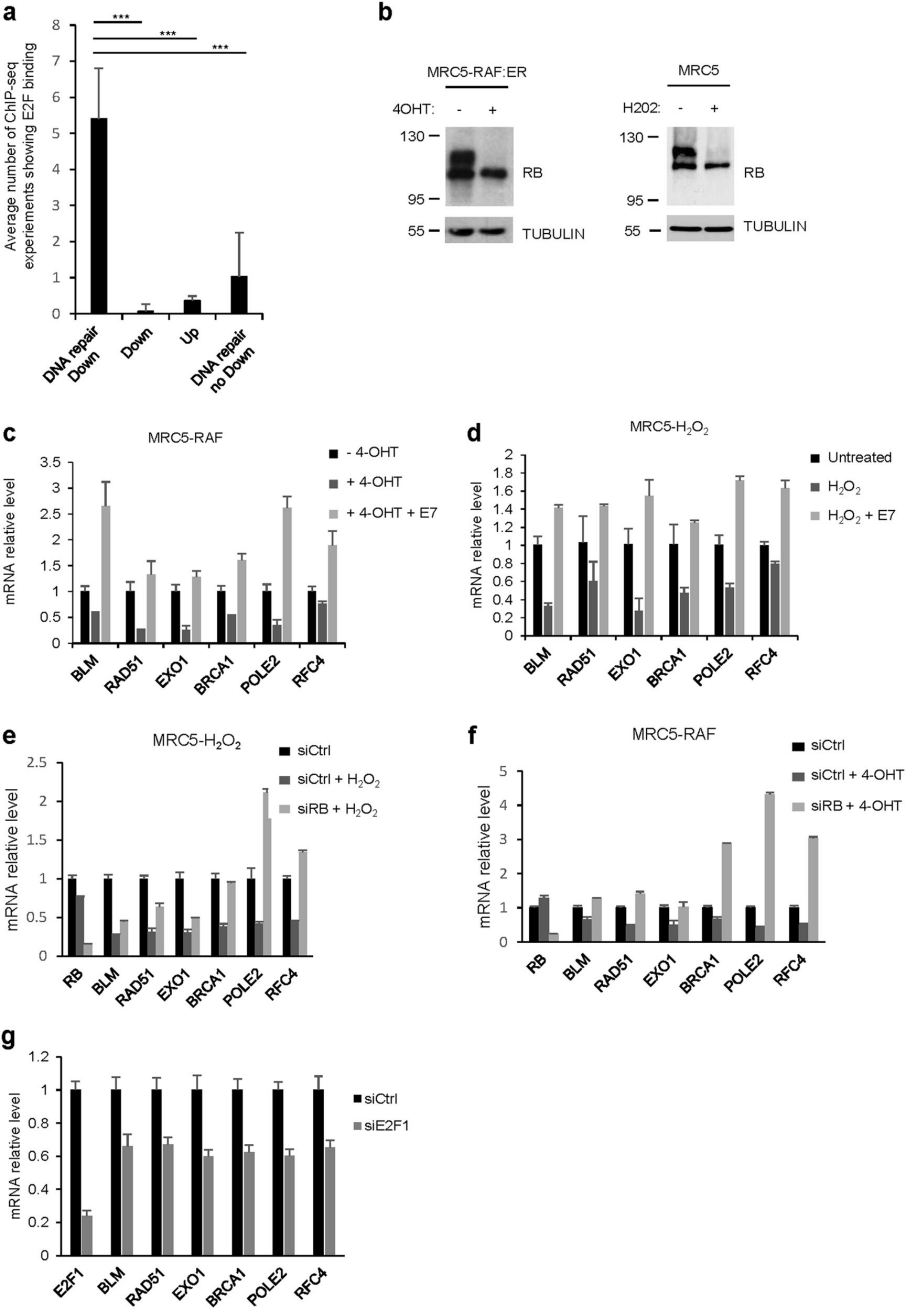
RB pathway mediates repression of DNA repair genes in senescent cells. **a** The transcription factor ChIP-seq uniform peaks database from the *Encode* consortium was explored for 7 ChIP-seq experiments performed on E2F1 or E2F4 transcription factors. We counted the number of experiments showing a ChIP-seq peak with a minimal score of 500 in the promoter of the top30 genes identified in transcriptome analysis of HMEC-MEK in four different classes: downregulated genes belonging to the GO DNA repair (DNA repair down); downregulated genes without GO enrichment (Dw); upregulated genes (Up); DNA repair genes not downregulated (DNA repair no down). *P*-values were determined using the student’s *t*-test. ***P < 0.001. **b** MRC5-RAF or MRC5 were treated with 4-OHT for 48 h or with H_2_O_2_ for 24 h. Cell lysates were prepared and analyzed by western blot with an antibody targeting RB. Tubulin was used as loading control. **c**–**f** MRC5-RAF or MRC5 cells were either infected with Ctrl or an E7-encoding retroviral vector or transfected with control siRNA (siCtrl) or siRNA directed against RB (siRB). Cells were treated with 4-OHT for 48 h or with H_2_O_2_ for 24 h. RT-qPCR for the indicated DNA repair genes were then performed. Results were normalized against GAPDH levels. Means ± SD are presented in the graph. A statistically significant down-regulation and reversion were observed for all the genes described (*t*-test *P* value <0.01). **g** MRC5 cells were transfected with a control siRNA (siCtrl) or with a siRNA directed against E2F1 (siE2F1). Three days after, RNA were prepared and RT-qPCR for the indicated genes were performed. Results were normalized against GAPDH levels. The different experiments shown are representative of at least two repeats. Statistically significant variations were observed for all the genes indicates (*P* < 0.01)

RB and E2F transcription factors are both master regulators of cellular senescence and the expression of DNA repair genes^23^. Therefore, we examined whether this pathway could regulate DNA repair gene repression during senescence. Interestingly, ChIP-seq experiments (*Encode—The Encyclopedia of DNA Elements*) demonstrated a strong enrichment in E2F bindings in the set of “repressed DNA repair genes” that we identified in HMEC-MEK cells compared to “downregulated non DNA repair genes”, “upregulated genes”, or “non-repressed DNA repair genes” sets (Fig. 2a and Supplementary Table S1). This is compelling with previous reports showing a direct regulation of DNA repair genes by E2F transcription factors^24,25^. Furthermore, western blot analysis revealed that RB protein was hypo-phosphorylated, a form that sequester and inhibit E2F factors, in senescing MRC5 fibroblasts (Fig. 2b). These results strongly support a role for the RB/E2F pathway in mediating DNA repair gene repression during senescence. To functionally confirm the involvement of the RB/E2F pathway, we inhibited RB by either stably expressing E7 viral protein^26,27^ or using siRNA directed against RB (siRB). Importantly, inhibition of RB abrogated DNA repair gene repression during senescence (Fig. 2c–f). In addition, the knock down of E2F1 was also sufficient to decrease DNA repair gene expression (Fig. 2g). These latter results substantiate the conclusion that RB, through E2F transcription factor, mediates repression of DNA repair genes in senescent cells.

### A single DNA repair gene loss-of-function induces features of premature senescence

**Fig. 3:**
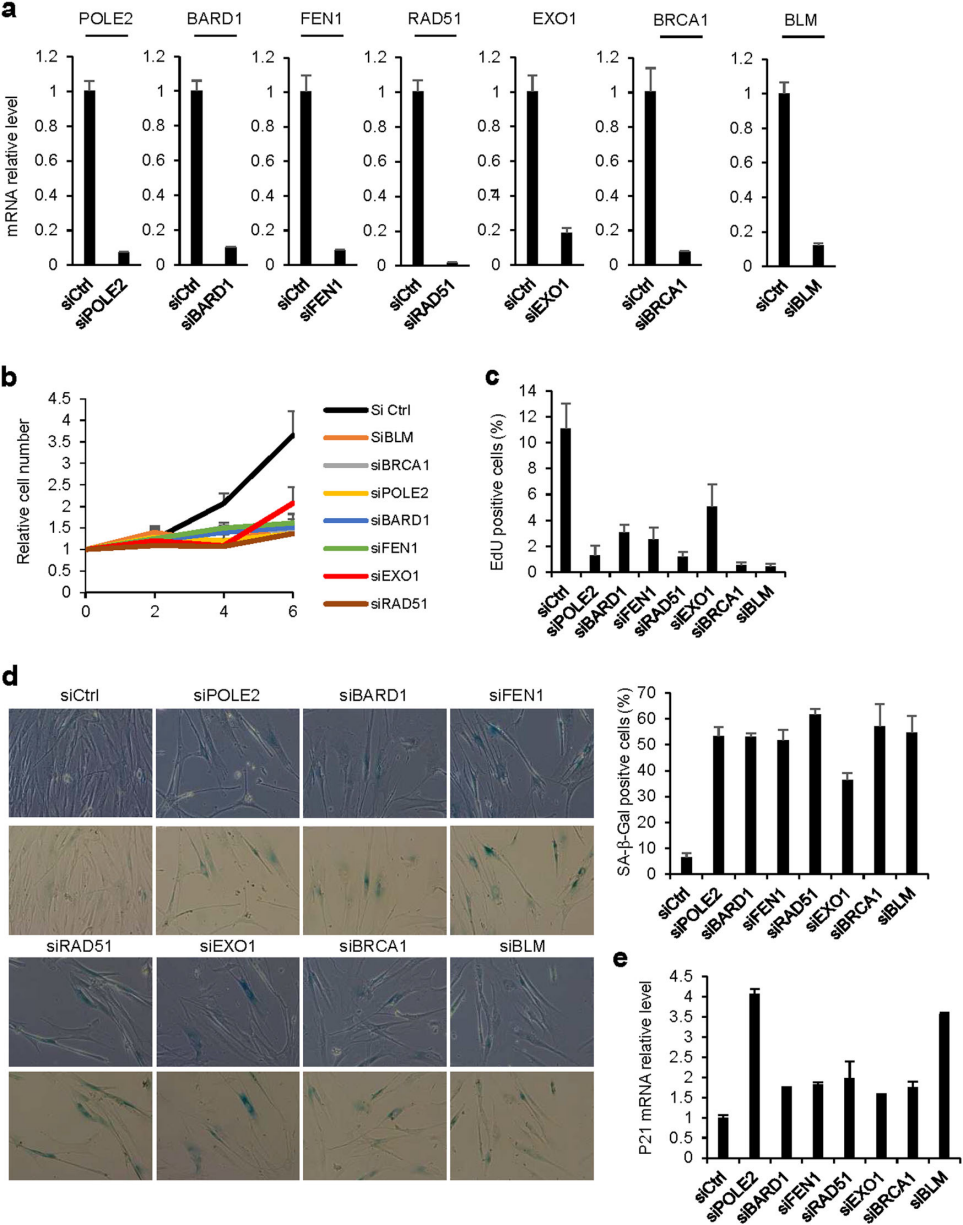
Loss-of-function of DNA repair genes leads to features of premature senescence. MRC5 cells were transfected with Ctrl siRNA or siRNA targeting the indicated DNA repair genes. Different assays were performed 5 days after transfection unless indicated otherwise. **a** RNAs were prepared and knock-down efficiency was verified by RT-qPCR after GAPDH normalization. Means ± SD are presented in the graph. A statistically significant knock-down was observed with the different siRNAs tested (*t*-test *P*-value <0.001). **b** Cells were seeded and counted every 2 days. Means ± SD are presented in the graph. A statistically significant difference in cell number observed between the Ctrl and the different siRNAs tested at d4 and d6 (*t*-test *P* value <0.01). **c** After 3 h incubation with EdU, the cells were fixed, stained and the percentage of EdU positive cells were counted. Means ± SD are presented in the graph. A statistically significant reduction of EDU positive cells was observed with all the siRNA against DNA repair genes tested (*P* < 0.01). **d** Cells were fixed and SA-β-Gal activity was assayed. Representative pictures and a graph showing the percentage of SA-β-Gal positive cells are presented. Means ± SD are presented in the graph. A statistically significant increase in percentage of SA-β-Gal positive cells was observed with the different siRNAs tested (*t*-test *P* < 0.001). (**e**) RT-qPCR against P21 was performed and results were normalized against GAPDH levels. A statistically significant of P21 was observed with the different siRNAs tested (*t*-test *P* < 0.05). The different experiments shown are representative of at least two repeats

The results presented above demonstrate that a vast program repressing DNA repair gene expression through the RB/E2F pathway occurs during cellular senescence. We then investigated whether this program is instrumental in the establishment of senescence. To this end, we tested several DNA repair genes by knocking-down their expression in human fibroblasts (Fig. 3a and Supplementary Figure S4a). Strikingly, knock-down of POLE2, BARD1, FEN1, RAD51, EXO1, BRCA1, or BLM alone was able to induce hallmarks of premature senescence. Indeed, their knock down (i) decreased cell proliferation according to the growth curves (Fig. 3b), to the stability of the arrest when cells were passaged (Supplementary Figure S4b), and the lack of increased number of dead cells (Supplementary Figure S4c), (ii) led to the decreased percentage of cells in S-phase according to EdU incorporation assays (Fig. 3c), (iii) increased the percentage of SA-β-Gal positive cells, together with increased cell size which is an additional hallmark of the senescent cells (Fig. 3d), (iv) and increased P21 senescence marker mRNA levels (Fig. 3e). To exclude any off target effects, we repeated these experiments using two independent siRNA against BRCA1 or BLM. We observed the same results confirming the specificity of the effects (Supplementary Figure S4d–g). Taken together, our data demonstrate that loss-of-function of a single DNA repair gene promotes features of premature senescence in normal human cells.

### Decreased expression of a single DNA repair gene induces DNA damage through repression of other DNA repair genes

**Fig. 4:**
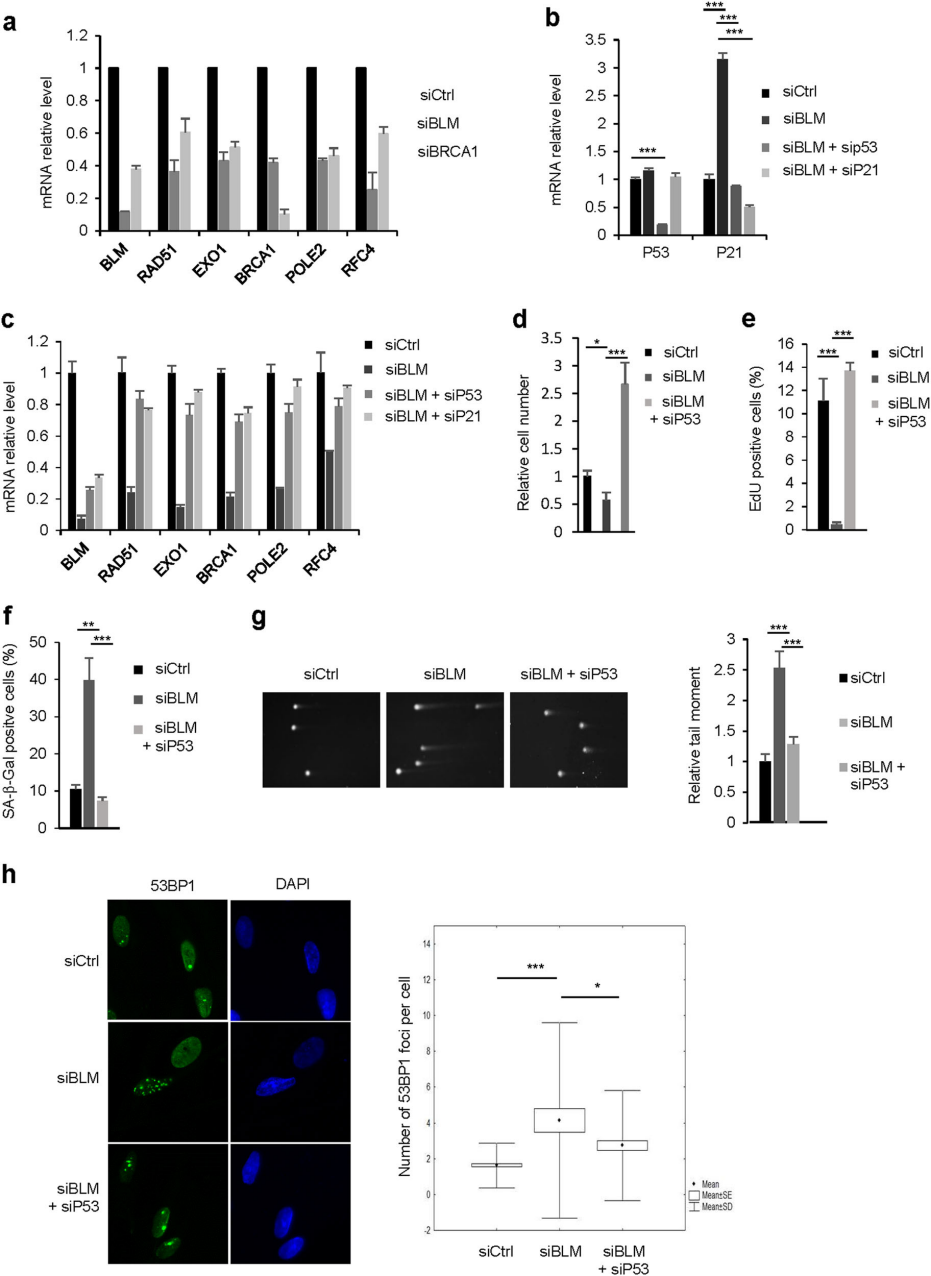
Decreased expression of BLM results in increased DNA damage through repression of other DNA repair genes and senescence. **a** MRC5 cells were transfected with a Ctrl, BLM or BRCA1 directed siRNA. After five days RNAs were prepared and levels of the indicated transcripts analyzed by RT-qPCR. GAPDH levels were used for normalization. Means ± SD are presented in the graph. A statistically significant downregulation was observed for all the genes described (*t*-test *P* < 0.001). **b**–**h** MRC5 cells were transfected with a Ctrl siRNA, a BLM siRNA or a combination of BLM and P53 or P21 siRNA as indicated, and different assays were performed after five days. **b**–**c** RNA were prepared and analyzed as in (**a**) for the indicated genes. Means ± SD are presented in the graph. A statistically significant downregulation and reversion were observed for all the genes described (*t*-test *P* < 0.001) (**c**). **d** Cells counts were performed and the relative cell number is shown. Means ± SD are presented in the graph. **e** After 3 h incubation with EdU, the cells were fixed, stained and the percentage of EdU positive cells counted automatically. Means ± SD are presented in the graph. **f** Cells were fixed and SA-β-Gal activity was measured. Means ± SD are presented in the graph. **g** Comet assays were performed and tail moments of 100 cells were quantified. Means ± SEM are presented in the graph. **h** Immunofluorescence detection of 53BP1 foci was performed. The number of 53BP1 foci per cell was scored using Focinator. The different experiments shown are representative of at least two repeats. *P*-values were determined using the student’s *t*-test. *P < 0.05; **P < 0.01; ***P < 0.001

Having shown that repression of DNA repair gene expression is associated with cellular senescence and that a loss-of-function of a single DNA repair gene favors features of premature senescence, we wondered whether loss-of-function of a single DNA repair gene also results in the repression of other DNA repair genes. For this purpose, we knocked-down BRCA1 or BLM. Strikingly, knock-down of BRCA1 or BLM was sufficient to repress other DNA repair genes (Fig. 4a), confirming that, even in this particular context, repression of DNA repair genes occurs during cellular senescence.

These results raised several questions: does repression of numerous DNA repair genes in response to the loss of a single DNA repair gene involve the P53/P21/RB pathway? Will it result in the induction of cellular senescence and of DNA damage? To address these issues we knocked-down P53, which subsequently led to the repression of P21 (Fig. 4b), knocked down P21 or RB. Strikingly, siRNA against P53, P21 or RB abolished the repression of DNA repair genes induced by the loss of BLM expression (Fig. 4c and Supplementary Figure S5a). In addition, inhibition of P53 blocked hallmarks of BLM siRNA-induced senescence. Indeed, P53 knock down increased the number of cells (Fig. 4d), without impacting cell death (Supplementary Figure S5b), it increased the proportion of cells incorporating EdU (Fig. 4e) and it decreased the percentage of SA-β-Gal positive cells (Fig. 4f) in the siBLM cells. Concomitantly, the siP53 reverted DNA damage accumulation induced by loss of BLM as evidenced by the decreased tail moment in the Comet assay (Fig. 4g) and by the decreased number of 53BP1 foci (Fig. 4h). The same observations were made when BRCA1 was knocked-down (Supplementary Figures S6).

Hence, these results support a role for decreased DNA repair gene expression in mediating DNA damage and senescence after initiation of the DNA damage process, by the loss of a single DNA repair gene.

### Activation of P53 and RB pathways is sufficient to repress DNA repair gene expression and promote DNA damage and its signaling

**Fig. 5:**
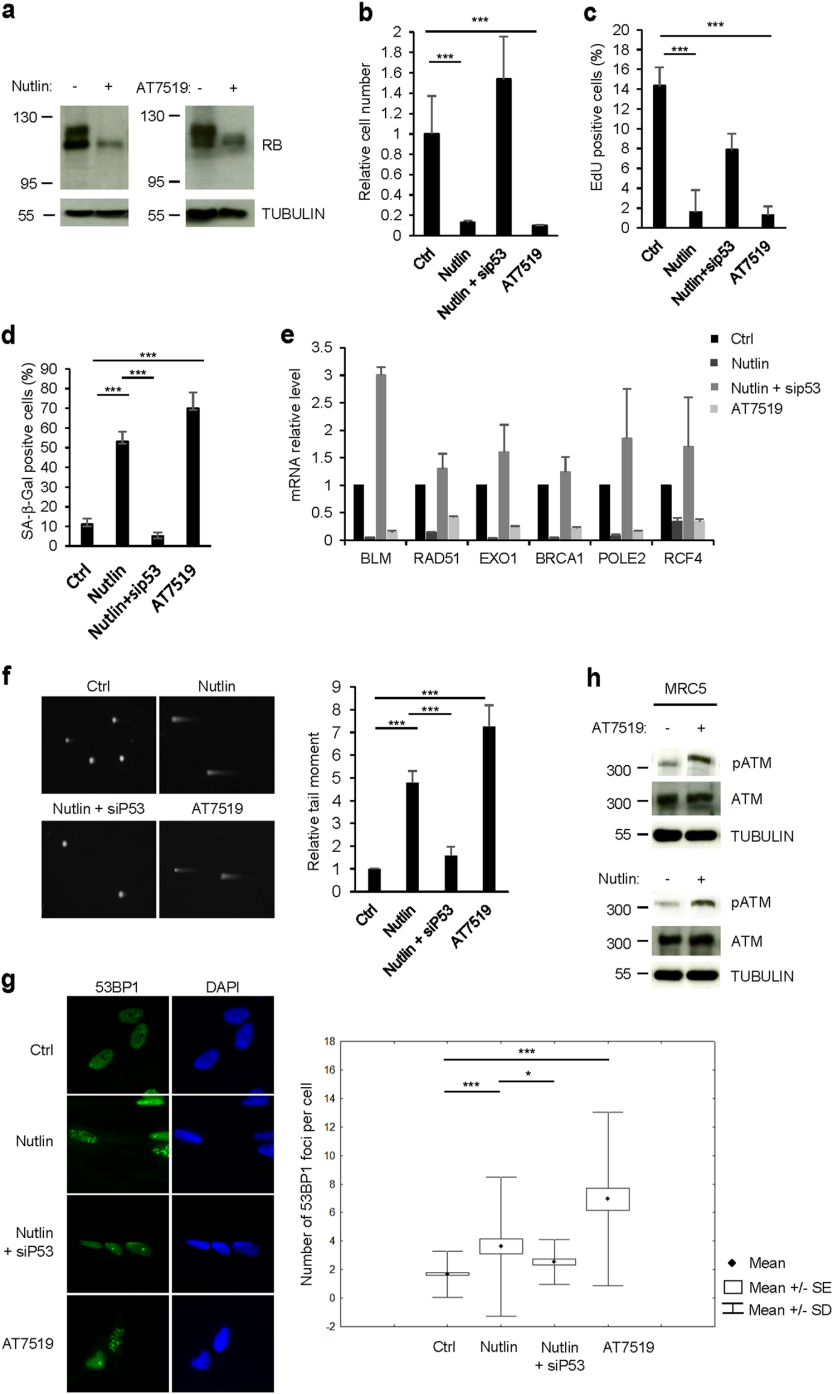
P53/RB activation and repression of DNA repair genes is sufficient to promote DNA damage accumulation and its signaling. MRC5 cells were treated either with the P53 activator Nutlin-3 (10 µM) or with the multi-CDK inhibitor AT7519 (0.5 µM). **a** Three days after treatment, western blot were performed against RB. Tubulin was used as loading control. **b** Three days after the indicated treatments, cells were counted. Means ± SD are presented in the graph. **c** Three days after the indicated treatment, MRC5 cells were incubated with EdU during 3 h and next cells were fixed and stained. Percentage of EdU positive cells was counted automatically. Means ± SD are presented in the graph. **d** Three days after the indicated treatments, cells were fixed and SA-β-Gal activity was assayed. Means ± SD are presented in the graph. **e** Twenty-four hours after treatment, RNAs were extracted and RT-qPCR against the indicated DNA repair genes performed. Results were normalized against GAPDH levels. Means ± SD are presented in the graph. A statistically significant down-regulation was observed for all the genes described (*t*-test *P* < 0.001). **f** Comet assays were performed 3 days after the indicated treatment. Tail moments of 100 cells were quantified. Means ± SEM are presented in the graph. **g** Three days after treatments, immunofluorescence against 53BP1 was performed. The number of 53BP1 foci per cell was scored using ImageJ. **h** Three days after the indicated treatment, cells were lysed and Western blot analysis was performed against pATM and ATM. Tubulin was used as loading control. The different experiments shown are representative of at least two repeats. *P*-values were determined using the student’s *t*-test. *P < 0.05; **P < 0.01; ***P < 0.001

As our previous results show that a P53/P21/RB pathway mediates DNA repair genes repression and promotes DNA damage accumulation upon knock down of BLM or BRCA1, we speculated that activation of RB by either constitutive activation of P53/P21 pathway or by constitutive inhibition of CDK/cyclin complexes, so without direct DNA lesions, should lead to the repression of the DNA repair gene expression, subsequently to inability to repair damaged DNA such as arising by collapsed replication forks or by oxidative stress, and then to the induction of a DNA damage response. To test this hypothesis, we activated P53 using Nutlin-3, an antagonist of MDM2^28^, and inhibited CDK/cyclin complexes using AT7519 inhibitor^29^. As expected, Nutlin-3 or AT7519 led to the accumulation of the hypophosphorylated form of RB (Fig. 5a) and to the induction of premature senescence in normal human cells, either MRC5 (Fig. 5b–d and Supplementary Figure S7a) or IMR90 (Supplementary Figures S8a–d). Furthermore, Nutlin-3 or AT7519 notably repressed DNA repair gene expression through a P53/P21/RB pathway (Fig. 5e and Supplementary Figures S7b-c and S8e)). This repression led to accumulation of DNA damage (Fig. 5f,g and Supplementary Figures S8f-g) and induced DNA damage signaling as measured by phosphorylation of ATM (Fig. 5h). Importantly, these data reveal that DNA damage and DNA damage response processes can be initiated by RB activation and without direct lesions to DNA.

## Discussion

In this study we unveiled the repression of numerous DNA repair genes as occurring during cellular senescence. Indeed, we observed a drastic decreased expression of DNA repair genes in every type of cellular senescence tested in vitro; oncogene-induced senescence, telomere-dependent senescence as well as oxidative stress-induced senescence, and in every cell type tested; fibroblasts, epithelial and endothelial cells. In few specific contexts of senescence, repression of DNA repair gene expression has been observed^23,30–32^, nevertheless this observation has never been extensively investigated as we did in our study with the conclusion that decreased DNA repair gene expression is associated with cellular senescence. The RB/E2Fs pathway has already been described to control the expression of numerous DNA repair genes^24,25^, and our results confirm the involvement of increased RB and subsequent decreased E2F activity in mediating the repression of DNA repair genes during senescence.

**Fig. 6:**
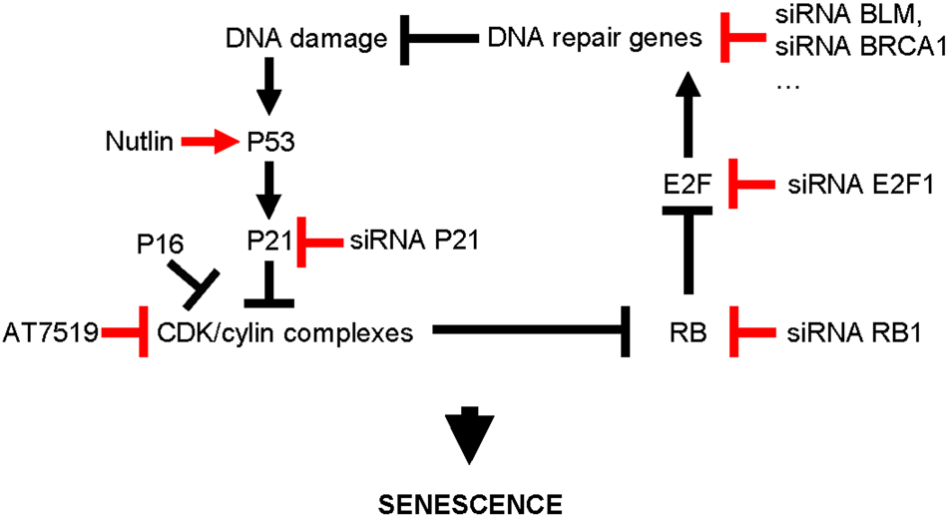
Model of amplifying loop leading to senescence. Schematic diagram depicting the new model in which (**i**) the repression of DNA repair genes would play a key role in senescence promotion through DNA damage accumulation and (**ii**) an amplification loop, in which the main actors are displayed, regulate senescence

DNA damage has been extensively observed in senescent cells and has thus been proposed to be a hallmark of senescent cells. So far, it has been assumed that this increased DNA damage in senescent cells results from either a direct attack of DNA by ROS, by IR or UV, by telomere shortening or by replicative stress upon oncogenic activation^13–16^. Our results further complexify these mechanisms as DNA damage and its signaling are observed when DNA repair gene expression is repressed by either loss of a single DNA repair gene like BRCA1 or BLM or by the sole activation of the P53-P21-RB pathway (Fig. 6). Since the decreased DNA repair gene expression is observed during OIS or ROS-induced senescence, we speculated that the decreased ability of cells to repair DNA contributes to the accumulation of DNA damage, which can be initiated for example by collapsed replication forks or by oxidative stress, in these systems. We thus propose that the decreased expression of DNA repair genes, from partly to completely, may explain increased DNA damage observed in senescent cells.

Repression of DNA repair genes has been observed in senescent cells and is thus a shared characteristic of senescent cells. Our results support a functional role of this repression in establishing the senescent phenotypes for several reasons: (i) repression of DNA repair genes occurs early and before establishment of senescent phenotypes (data not shown) and (ii) the knock-down of every DNA repair gene tested promotes features of cellular senescence. Consistently, loss-of-function of some DNA repair genes has already been implicated in induction of senescence, while gain-of-function of some DNA repair genes was shown to inhibit senescence in some specific contexts^30,33–35^. DNA repair gene repression is thus a key inducer of cellular senescence (Fig. 6).

Loss-of-function of DNA repair genes, such as BRCA1 or BLM, has been linked to premature aging and/or to the predisposition of cells to form tumors, and chronic premature senescence is thought to participate in both^6,36–40^. Our results highlight that features of premature senescence observed upon loss of some of these genes are mediated by repression of numerous other DNA repair genes through a P53/P21/RB pathway as their knockdown reverts this repression. This supports the view that even if the initial event is different (deficiency in BRCA1, BLM or others), the loss-of-function of different DNA repair genes converge towards the activation of the P53/P21/RB pathway to mediate repression of numerous DNA repair genes. As in normal cells, DNA damage occurs throughout the cell cycle, arising from collapsed replication forks or oxidative stress for example, the inability to repair DNA can result in increased DNA damage and subsequent induction of senescence (Fig. 6). We thus propose a novel way of envisaging how mutation/loss-of-function of some DNA repair genes may promote a broad repression of DNA repair genes, DNA damage accumulation, and genomic instability to favor both aging and cancer.

One of the most striking results of this study is that the sole activation of the P53-P21-RB pathway by the small molecules Nutlin, to activate P53, or AT7519, to inhibit the CDK/cylin complexes, was able to induce (i) DNA repair genes repression, (ii) DNA damage accumulation, (iii) and senescence (Fig. 6). Specificity of the effects caused by Nutlin was confirmed using siRNA against P53 and similar results were obtained in MRC5 and IMR90 normal human cells. Although the results observed appear to be controversial, as Nutlin does not have direct genotoxic effects^41^, a similar finding, i.e., induction of DNA damage by Nutlin through P53, has already been reported^42^. Besides these in vitro results, increased p53 activity in mice expressing the p53Δ31 transgene also leads to DNA repair gene repression, at least for Fanconi Anemia DNA repair pathway, through the E2F transcription factors^43^. Therefore in addition to the pivotal role of the tumor suppressor P53 in the DNA damage response, we suggest that sustained activity of P53 could favor accumulation of DNA damage through DNA repair genes repression and subsequently lead to genetic instability in the context of senescence, which might contribute to premature aging, as previously described in different mouse models^44,45^.

In conclusion, our in vitro results delineate repression of DNA repair genes as occurring in senescent cells and show that this repression is both important for induction of DNA damage and induction of cellular senescence through an amplifying loop (Fig. 6). These results offer a novel perspective on the role of DNA repair gene repression on senescence, cancer and aging.

## Materials and methods

### Cell culture and reagents

Primary human mammary epithelial cells (HMECs) were provided by Lonza. HMECs were cultured in mammary epithelial cell growth medium (Promocell) with penicillin/streptomycin 100 U/mL (Life Technologies). Human fetal lung fibroblasts MRC5 and IMR90 (ATCC) and virus-packaging cells GP293 (Clontech) were cultured in DMEM Medium (Life Technologies) supplemented with 10% fetal bovine serum (Life Technologies) and penicillin/streptomycin 100 U/mL. The cells were maintained at 37 °C under 5% CO_2_ atmosphere. HMEC, MRC5 and IMR90 express a wild type P53.

Selection was done with puromycin (Invivogen) at 0.5 µg/mL, geneticin (Life Technologies) at 100 µg/mL or hygromycin (Invitrogen) at 100 µg/mL. AT7519 (Selleckchem) was used at 0.5 µM. Nutlin-3 (Sigma Aldrich) was used at 10 µM. H_2_0_2_ was added at 250 µM during 1 h. 4-Hydroxytamoxyfen (4-OHT, Sigma Aldrich) was used at 100 nM for activation of RAF or MEK oncogene.

### Vectors, transfection, and infection

pBABE-hygro-hTERT (Addgene plasmid #1773) was used to immortalize HMECs. pNLCΔMEK1 (ΔN3, S218E, S222D):ER was used to transfer the MEK oncogene into HMECs. pBabe/RAF:ER^46^ was used to transfer RAF into MRC5 cells. pLXSN/E7^47^ was used to transfer E7 into MRC5. pWZL Hygro-TRF2 deltaB deltaM (Plasmid #18013) was used to transfer ΔTRF2 into MRC5.

Virus producing GP293 cells were transfected with retroviral vector of interest in combination with the VSVg using the PEI reagent (Euromedex) as previously described^48^. Two days after transfection, the viral supernatant mixed with fresh medium (1 of 2) and hexadimethrine bromide at 8 μg/mL (Sigma) was used to infect target cells. Cells were infected for 6 h and selected the day after infection using hygromycin for ΔTRF2, geneticin for MEK:ER or E7 and puromycin for RAF:ER.

### siRNA transfection

DharmaFECT 1 small interfering RNA (siRNA) transfection reagent, siGENOME SMARTpool siRNAs for the targeted genes (as mentioned in the figures), and siGENOME RISC-free control siRNAs, named siCtrl, were purchased from Dharmacon. MRC5 cells were reverse transfected using 30 nM siRNA in 6-well plates (5 × 10^5^ per well), using a 0.1% solution of Dharmafect 1 Transfection reagent (Thermo scientific). The sequences of siRNAs for BLM#1, BLM#2, BRCA1#1 and BRCA1#2 were as follows: BLM#1 (5′-CUAAAUCUGUGGAGGGUUA-3′), BLM#2 (5′-GCAACUAGAACGUCACUCA-3′), BRCA1#1 (5′-UAUCAUCGCCCAUGCAUCA-3′) and BRCA1#2 (5′-CUAAUCAGGUGGUAGCUCA-3′).

### Cell count and EdU staining

Two thousand cells per well in triplicates were plated in Clear Advanced TC 96-well microplate (Greiner) and treated with Nutlin, AT7519, or the different siRNA for 3 days. Then cells were exposed to 10 μM EdU for 3 h prior to fixation with 3.7% paraformaldehyde in PBS at room temperature for 15 min. Cells were permeabilized with 0.5% Triton X100 in PBS for 20 min at room temperature. EdU was labeled with AF488 using a Click-iT EdU labeling kit (C10337, LifeTechnologies) as indicated by the manufacturer. DNA was co-stained with Hoechst 33342 (1 μg/mL) at room temperature for 30 min and washedwith PBS. Atleast 10 fields were imaged with Operetta high-content imaging system (PerkinElmer) at 10× magnification and they were analyzed with the Columbus (PerkinElmer) software to calculate the relative cell number or/and the percentage of EdU positive cells.

### SA-β-Gal analysis and crystal violet staining

For SA-β-Gal staining, cells were fixed for 4 min in 2% formaldehyde/0.2% glutaraldehyde, washed twice with PBS and incubated overnight at 37 °C in SA-β-Gal staining solutions as previously described^49^. For crystal violet staining, cells were fixed for 10 min in 4% formaldehyde, washed twice and counterstained with 0.05% crystal violet (Sigma-Aldrich).

### Transcriptome and bioinformatics analysis

Transcriptome analysis of HMEC-MEK, treated or not with 100 nM 4-OHT to induce MEK expression, were performed using Whole Human Genome Oligo 4 × 44 K Microarrays (Agilent Technologies) and the one-color gene expression Agilent workflow. Briefly, cRNAs were synthesized and labeled with the Cy3 dye from 100 ng of total RNA using the one-color Low Input Quick Amp Labeling Kit (Agilent Technologies). Then, 1650 ng of Cy3-labeled cRNAs purified using the RNeasy Mini-spin columns (Qiagen) were hybridized on to the 4 × 44 K arrays for 17 h at 65 °C. Microarrays were washed and scanned with an Agilent DNA microarray scanner G2565CA (Agilent Technologies). Fluorescent signals were extracted and normalized using the Feature Extraction Software Version 10.5.1.1 (Agilent Technologies), then transferred to the Genespring GX 12.6 software (Agilent Technologies) for data processing and data mining. All of the conditions were tested in three independent biological replicates for statistical analyses. Microarray probes were filtered using the Agilent flag filter to remove probes with a raw signal below 30 in at least one of the conditions tested. Genes differentially expressed between 4-OHT treated and untreated HMEC-MEK cells were defined using an unpaired *t*-test *P*-value <0.01 with a Benjamani Hochberg correction and fold change cutoffs > or <2, for upregulation and downregulation, respectively. The GO tool from *GeneSpring* enabled us to determine statistically significant enrichments in biological processes, based on computation *P*-values described by standard hypergeometric distribution.

### Reverse transcription and real-time quantitative PCR

Total RNA was isolated with a phenol-chloroform extraction method, using TriReagent (Sigma-Aldrich). Then, 1 µg of total RNA was reverse-transcribed using the Dynamo cDNA Synthesis Kit (Fisher Scientific) according to the manufacturer’s instructions. 1:10 dilution of this RT reaction mixture was used as the cDNA template for qPCR. TaqMan quantitative PCR analysis was carried out using the CFX96 Connect Real-Time PCR Detection System (Bio-Rad). The FastStart Essential Probes Master (Roche) was used as PCR mix. Human *GAPDH* was used for normalization. The primers and probes used are listed in the Supplementary Table S2.

### Immunofluorescence

MRC5 cells were grown in 8 chamber tissue culture glass slides (Falcon, Corning). After the indicated treatments, cells were fixed in ice-cold methanol for 10 min at −20 °C, and blocked in PBS-Tween 0.01% containing 1% Bovine Serum Albumin (PBST-BSA) for 2 × 15 min. Incubation with the 53BP1 antibody (dilution 1:300, Cell Signaling Technology, #4937) in PBST-BSA was performed overnight at 4 °C. After three washes with PBS, the slides were incubated with Alexa Fluor 488 dye-conjugated goat anti-rabbit antibody diluted in PBST-BSA (dilution 1:500) for 1 h at room temperature. After three washes in PBS, the slides were then mounted with DAPI Fluoromount G (SouthernBiotech). Images were acquired with a Nikon fluorescence microscope, and data were collected and analyzed with NIS software (Nikon). The number of 53BP1 foci have been determined using the Focinator tool^50^.

### Immunoblot analysis

Cell lysates were prepared in 6 × Laemmli buffer. Protein expression was examined by western blotting using rabbit anti-RAD51 (1:500, sc-8349, Santa Cruz Biotechnology), anti-EXO1 (1:500, sc-19941, Santa Cruz Biotechnology), anti-RFC4 (1:500, ab182145, abcam), anti-FEN1 (1:500, sc28355, Santa Cruz Biotechnology), anti-BLM (1:500, sc365753), anti-tubulin (1:5000, T6199, and Sigma), anti-ATM (phospho S1981) antibody (1:1000, ab81292, and abcam), anti-ATM (1:1000, ab78, and abcam) and anti-RB (1:250, 554136, BD Pharmingen). Horseradish peroxidase-conjugated donkey antirabbit (Interchim), sheep anti-mouse antibodies (Interchim) or donkey anti-goat (Santa Cruz Biotechnology) were used as secondary antibodies. Protein bands were detected by Western blot using an ECL Detection Kit (Amersham).

### Comet assay

Cells were suspended 1:10 in 0.5% low-melting point agarose at 37 °C. The suspension was immediately poured onto a Comet slide (Trevigen Inc.) (2000 cells per well). Agarose was allowed to solidify at 4 °C for 15 min. The Comet slides were then immersed in pre-chilled lysis solution (1.2 M NaCl, 100 Mm EDTA, 10 mM Tris, 1% Triton (pH 10)) at 4 °C for 120 min in the dark. After this treatment, comet slides were allowed to equilibrate in electrophoresis buffer for 2 × 15 min at 4 °C. Migration was performed in EDTA 2 mM NaOH 30 mM (pH 12.3) buffer. After migration, the slides were rinsed with water, neutralized with 0.4 M Tris (pH 7.5), fixed 5 min in 70% ethanol and stained with SYBR Safe (X1000; Invitrogen) according to manufacturer’s recommendations. Images were acquired with a Nikon fluorescence microscope and NIS software (Nikon). Tail moments were analyzed by using the Casplab freeware.

### Statistical analysis

Graphs are presented with SD as errors bars, and Student’s *t*-test was used to determine the *P*-value. * *P* < 0.05; ** *P* < 0.01; *** *P < *0.001 unless specified otherwise in the figure legends. Boxplots were made with Statistica.

## Electronic supplementary material

Supplemental Figure Legends

Supplemental Figures

Supplemental Tables
