# Supplementary material for: Transcriptional repression of DNA repair genes is a hallmark and a cause of cellular senescence
Source: Cell Death Dis. 2018 Feb 15;9(3):259. doi: 10.1038/s41419-018-0300-z (PMC5833687; doi:10.1038/s41419-018-0300-z)
Supplement: Supplementary file 1 — Supplemental Figure Legends [file 41419_2018_300_MOESM1_ESM.docx]

**Supplemental Figure Legends**

**Figure S1** RAF expression induces senescence in MRC5 cells. Cells were infected with a RAF:ER encoding retroviral vector and puromycin selected. MRC5-RAF cells were seeded and treated with 4-OHT for 3 days to activate RAF. (**a**) Cells were fixed and stained with crystal violet to monitor cell density. (**b**) RNAs were extracted and RT-qPCR against the KI67 proliferation marker performed. Results were normalized against GAPDH levels. Means ± SD are presented in the graph. (**c**) Cells were fixed and SA-β-Gal activity analyzed. Means ± SD are presented in the graph. The different experiments shown are representative of at least 2 repeats. P-values were determined using the student’s t-test. ***P<0.001.

**Figure S2** H_2_O_2_ treatment induces senescence in MRC5 cells. Cells were seeded and treated with H_2_O_2_ for 1 h on the following day. Six days later different tests were performed. (**a**) Cells were fixed and crystal violet stained to monitor cell density. (**b**) RNAs were extracted and RT-qPCR against the KI67 proliferation marker performed. Results are normalized against GAPDH levels. Means ± SD are presented in the graph. (**c**) Cells were fixed and stained for SA-β-Gal activity. Means ± SD are presented in the graph. The different experiments shown are representative of at least 2 repeats. P-values were determined using the student’s t-test. ***P<0.001.

**Figure S3** ΔTRF2 expression induces senescence in MRC5 cells. Cells were infected with a ctrl or a ΔTRF2 encoding retroviral vector. Ten days after infection, senescence was assessed. (**a**) Cells were fixed and stained with crystal violet to assess cell density. (**b**) RNAs were extracted and RT-qPCR against the KI67 proliferation marker performed. Results were normalized against GAPDH levels. Means ± SD are presented in the graph. (**c**) Cells were fixed and SA-β-Gal activity was assessed. Means ± SD are presented in the graph. The different experiments shown are representative of at least 2 repeats. P-values were determined using the student’s t-test. ***P<0.001.

**Figure S4** Knock-down of DNA repair genes promotes hallmark of senescence. MRC5 cells were transfected with siRNA pools against the DNA repair genes indicated (**a-c**) or with individual siRNA against BLM (siBLM#1, siBLM#2) and BRCA1 (siBRCA1#1, siBRCA1#2) (**d-g**). (**a**) Cell lysates were prepared 4 days after transfection and analyzed by western blot using the indicated antibodies. Tubulin was used as loading control. (**b**) Three days after transfection, cells were counted and the same amounts of cells were seeded back. Four days later cells were counted and the relative cell number is displayed. Means ± SD are presented in the graph. (**c**) Five days after transfection, percentage of blue trypan positive cells were counted to determine the percentage of dead cells. Thapsigargin treatment (5 µM during the last 2 days) was used as a positive control for inducing cell death. Means ± SD are presented in the graph. (**d-g**) Five days after transfection, (d) RT-qPCR for the indicated DNA repair genes was performed. Results were normalized against GAPDH levels. Means ± SD are presented in the graph. (e) Cell number was calculated. Means ± SD are presented in the graph. (f) EdU was incubated during 3h and percentage of positive cells automatically counted. Means ± SD are presented in the graph. (g) Cells were fixed and SA-β-Gal activity was assayed. Means ± SD are presented in the graph. A statistically significant change was observed for all assays between ctrl and knock-down conditions (t-test P<0.01). The different experiments shown are representative of at least 2 repeats.

**Figure S5** BLM knock-down effects. MRC5 cells were transfected with a control siRNA (siCtrl) or with a BLM siRNA (siBLM) or with a combination of BLM siRNA and RB siRNA (siBLM + siRB). (**a**) Five days later, RT-qPCR against the indicated DNA repair genes was performed. Results were normalized against GAPDH levels. Means ± SD are presented in the graph. A significant down-regulation was observed for all the indicated DNA repair genes (t-test P<0.05) (**b**) Five days after transfection, percentage of blue trypan positive cells were counted to determine the percentage of dead cells. Thapsigargin treatment (5 µM during the 2 last days) was used as a positive control for inducing cell death. Means ± SD are presented in the graph. The different experiments shown are representative of at least 2 repeats.

**Figure S6** Decreased expression of BRCA1 results in increased DNA damage through the repression of other DNA repair genes and senescence. MRC5 cells were transfected with a control siRNA (siCtrl), or a BRCA1 siRNA (siBRCA1) or a combination of BRCA1 siRNA and P53 siRNA (siBRCA1 + siP53) or P21 siRNA (siBRCA1 + siP21) or RB siRNA (siBRCA1 + siRB). After 5 days different assays were performed. (**a**-**c**) RNA were prepared and relative expression was analyzed for the indicated genes. Results were normalized to GAPDH levels. Means ± SD are presented in the graph. For (**b** and **c**), a statistically significant down-regulation and reversion were observed for all the genes described (t-test P<0.001). (**d**) Cells counts were performed and the relative cell number is shown. Means ± SD are presented in the graph. Means ± SD are presented in the graph. (**e**) After 3h incubation with EdU, the cells were fixed, stained and the percentage of EdU positive cells counted automatically. Means ± SD are presented in the graph. (**f**) Cells were fixed and SA-β-Gal activity was measured. Means ± SD are presented in the graph. (**g**) Five days after transfection, percentage of blue trypan positive cells were counted to determine the percentage of dead cells. Thapsigargin treatment (5 µM during the 2 last days) was used as a positive control to induce cell death. Means ± SD are presented in the graph. (**h**) Comet assays were performed and Tail moments of 100 cells were quantified. Means ± SEM are presented in the graph. (**i**) Immunofluorescence detection of 53BP1 foci was performed. The number of 53BP1 foci per cell was scored using ImageJ. The different experiments shown are representative of at least 2 repeats. P-values were determined using the student’s t-test. *P<0.05; **P<0.01; ***P<0.001.

**Figure S7** Effect of Nutlin-3 and AT7519 on MRC5 cells. (**a**) MRC5 cells were treated either by Nutlin-3 or AT7519 for 3 days. Then the percentage of blue trypan positive cells was counted to determine the percentage of dead cells. Thapsigargin treatment was used as a positive control to induce cell death. Means ± SD are presented in the graph. (**b-c**) MRC5 cells were transfected with control siRNA (siCtrl), siRNA directed against P21 (siP21) or RB (siRB) and they were treated the next day by Nutlin-3 (5 µM). Three day later, RNAs were extracted and RT-qPCR performed against the indicated DNA. Results were normalized against GAPDH levels. Means ± SD are presented in the graph. The different experiments shown are representative of at least 2 repeats. A statistically significant change was observed for all the indicated DNA repair genes between ctrl and Nutlin or between Nutlin and Nutlin + siP53 (t-test P<0.05)

**Figure S8** P53/RB activation and repression of DNA repair genes is sufficient to promote senescence and DNA damage accumulation in IMR90 cells. IMR90 cells were transfected with a control siRNA or with a P53 directed siRNA. One day later, they were treated with Nutlin-3 (5 µM). (**a**) Three days after, cells were counted. Means ± SD are presented in the graph. (**b**) Three days after the indicated treatment, MRC5 cells were incubated with EdU during 3 h and next cells were fixed and stained. Percentage of EdU positive cells counted automatically. Means ± SD are presented in the graph. (**c**) Three days later, percentage of blue trypan positive cells was counted to determine the percentage of dead cells. Thapsigargin treatment (5µM during the 2 last days) was used as a positive control to induce cell death. Means ± SD are presented in the graph. (**d**) Three days later, cells were fixed and SA-β-Gal activity was assayed. Means ± SD are presented in the graph. (**e**) Twenty four hours after treatment, RNAs were extracted and RT-qPCR performed. Results were normalized against GAPDH levels. Means ± SD are presented in the graph. A statistically significant change was observed for all the indicated DNA repair genes between ctrl and Nutlin or between Nutlin and Nutlin+siP53 (t-test P<0.01). (**f**) Comet assays were performed 3 days after nutlin treatment. Tail moments of at least 100 cells were quantified. Means ± SEM are presented in the graph. (**g**) Three days after treatment, immunofluorescence against 53BP1 was performed. The number of 53BP1 foci per cell was scored using ImageJ. The different experiments shown are representative of at least 2 repeats. P-values were determined using the student’s t-test. *P<0.05; **P<0.01; ***P<0.001.
