# Supplementary figures and images for: Transcriptional repression of DNA repair genes is a hallmark and a cause of cellular senescence
Source: Cell Death Dis. 2018 Feb 15;9(3):259. doi: 10.1038/s41419-018-0300-z (PMC5833687; doi:10.1038/s41419-018-0300-z)

**Figure S1**

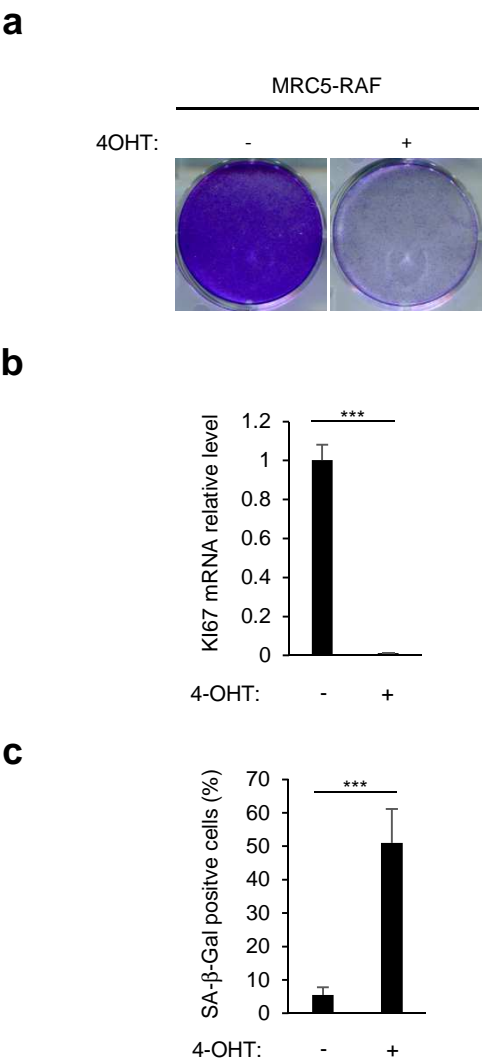

**Figure S2**

**a**

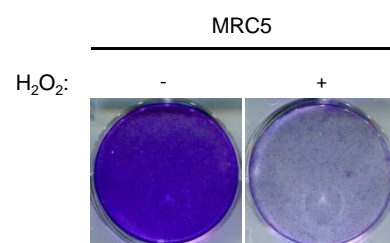

**b**

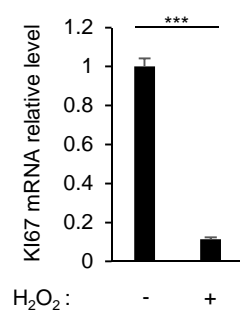

**c**

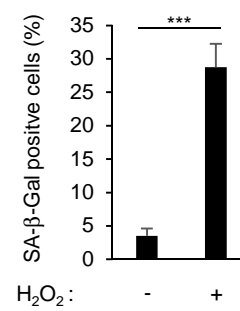

**Figure S3**

**a**

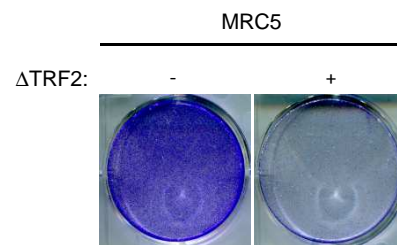

**b**

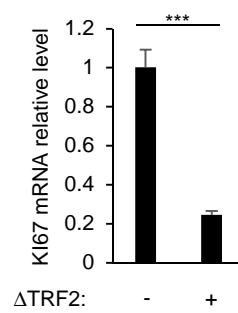

**c**

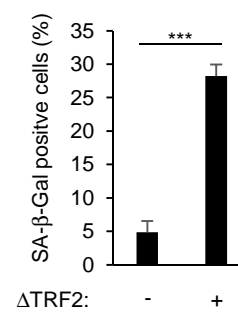

**Figure S4**

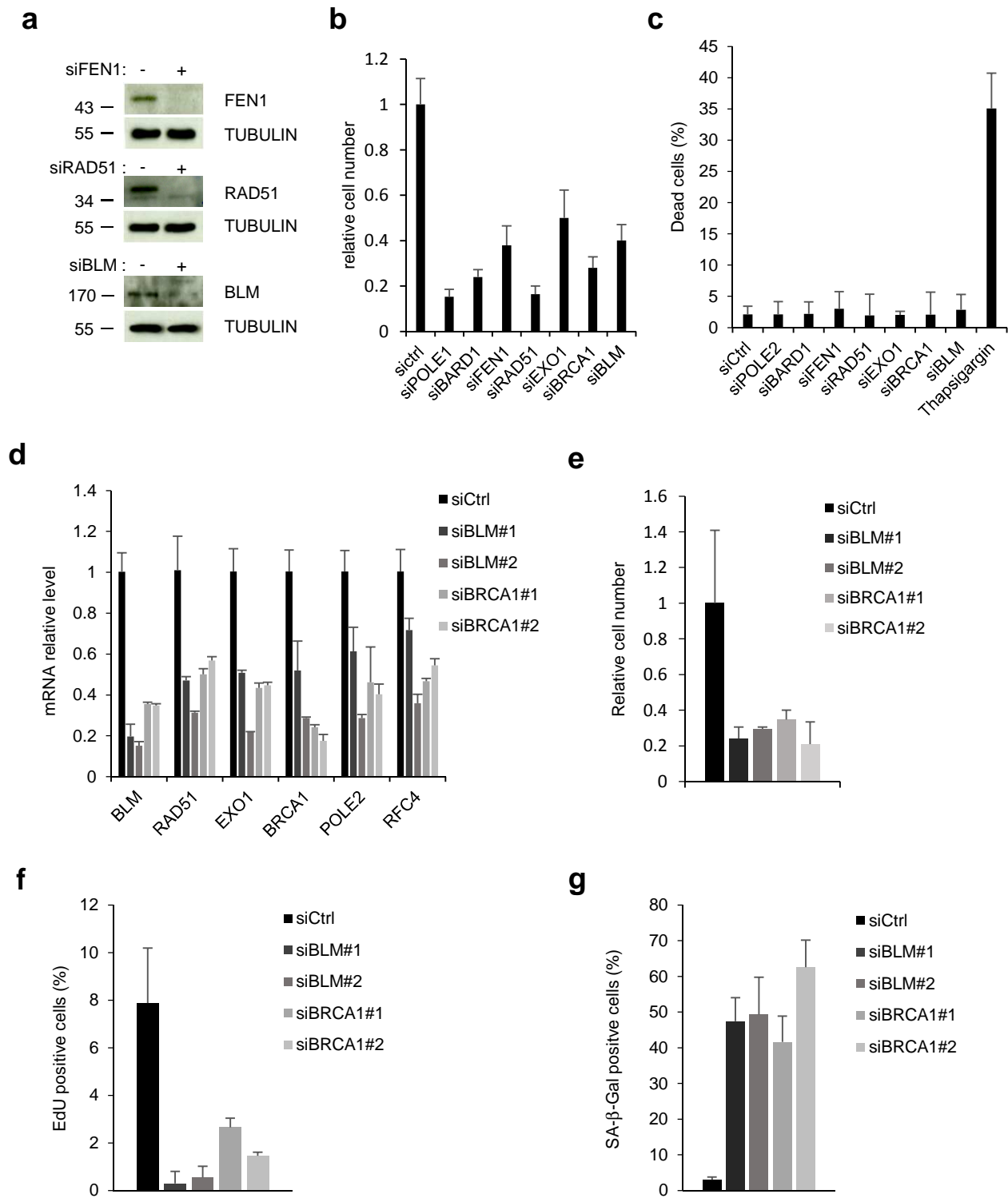

**Figure S5**

**a**

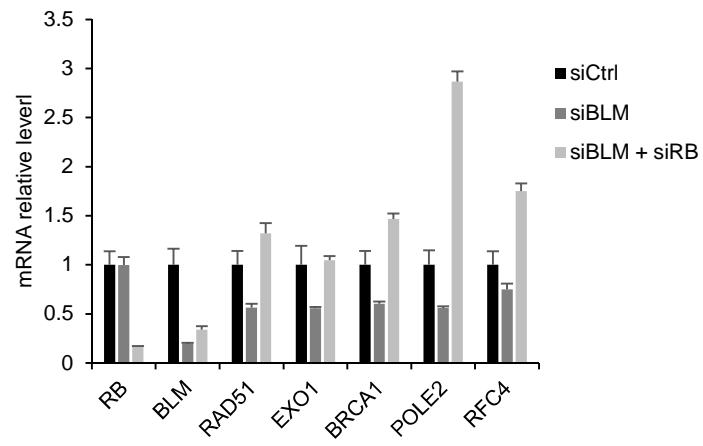

**b**

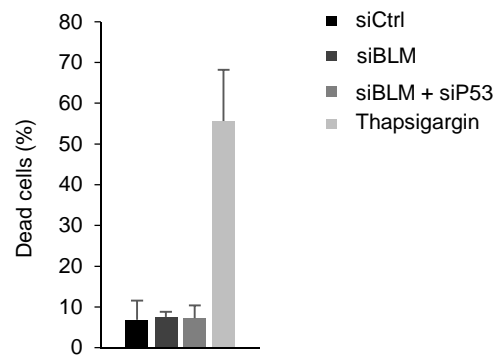

**Figure S6**

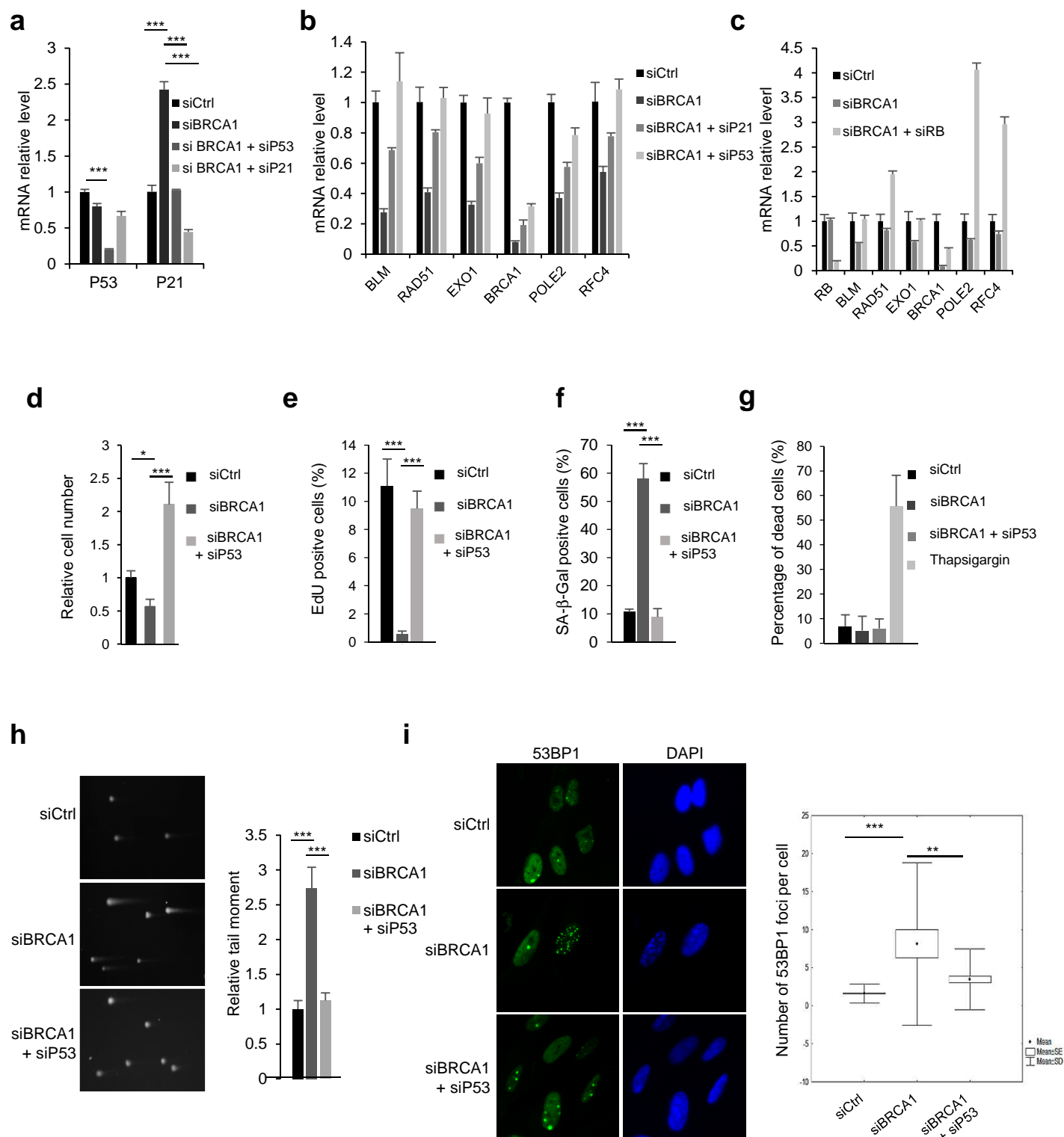

**Figure S7**

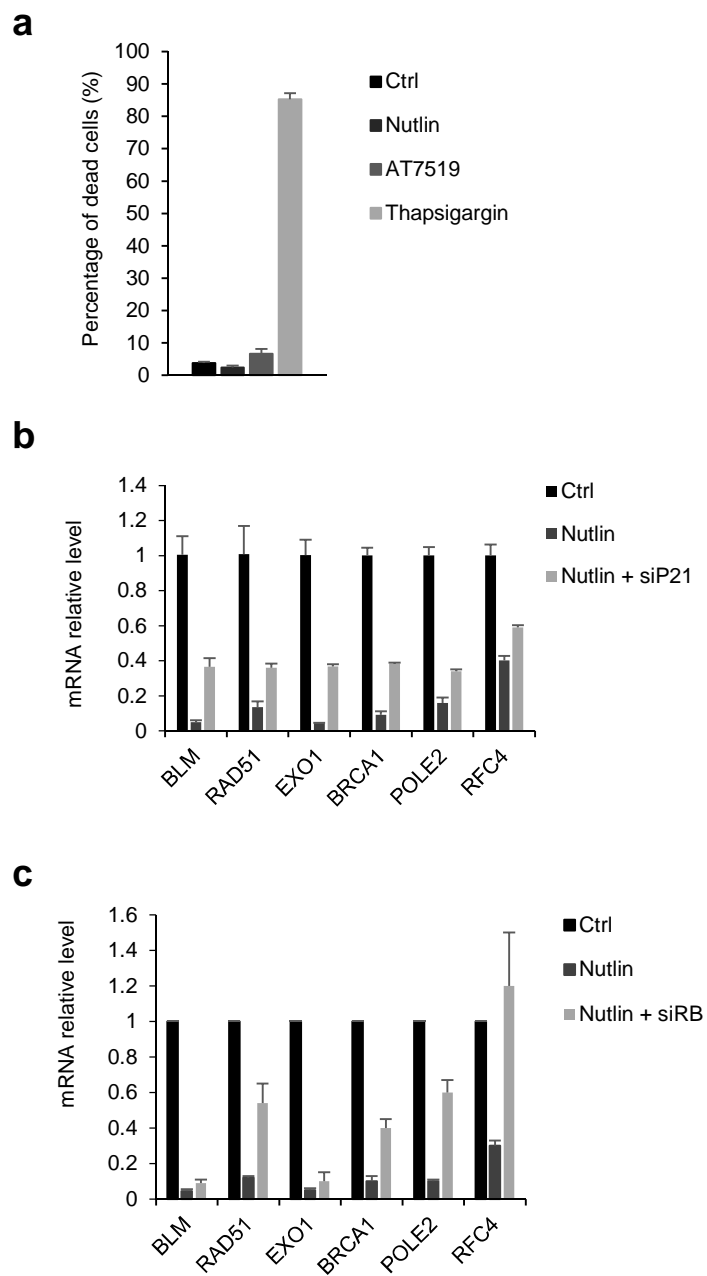

**Figure S8**

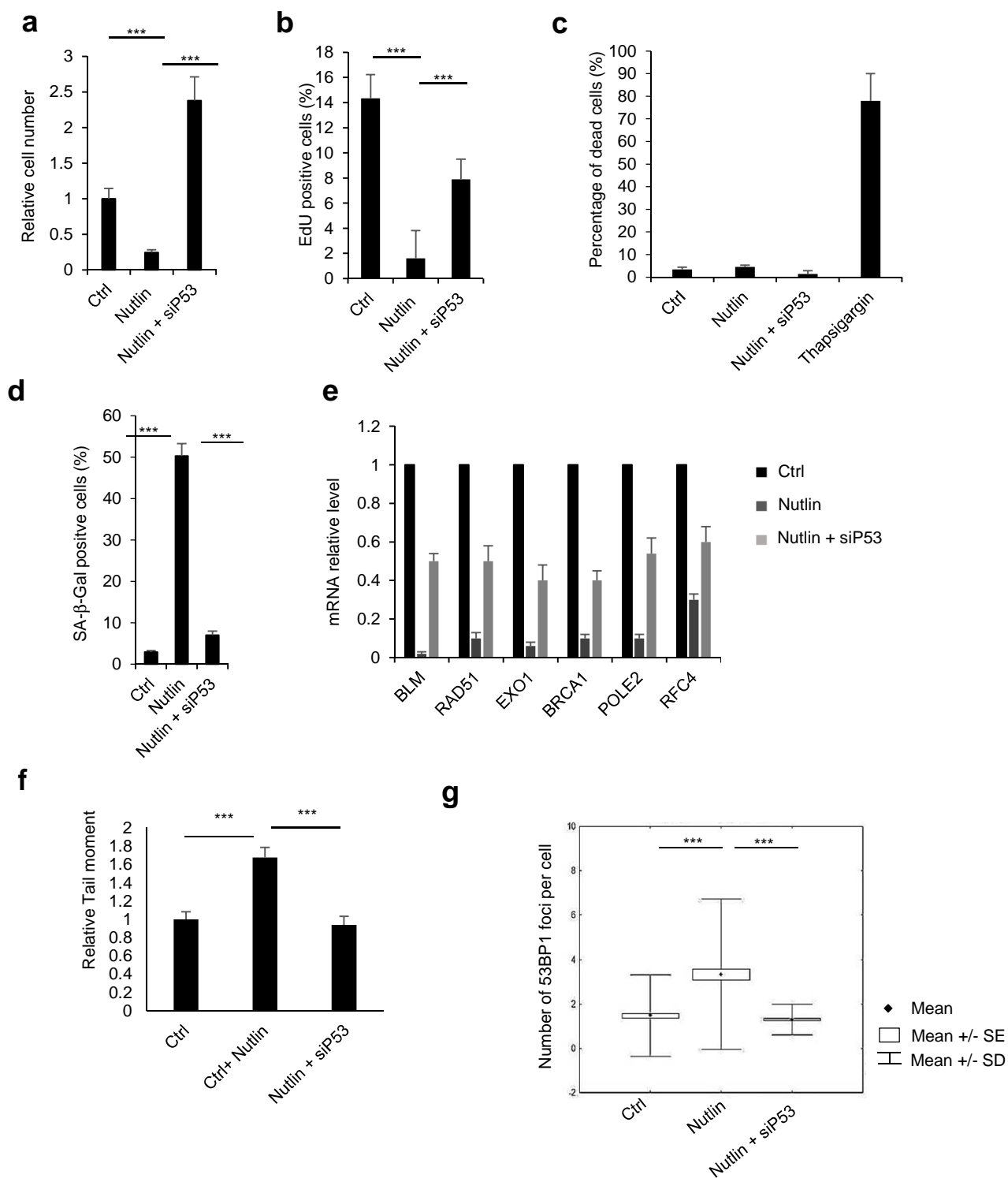

Supplement: Supplementary file 2 — Supplemental Figures [file 41419_2018_300_MOESM2_ESM.pdf]
