# Supplementary material for: Transcriptional repression of DNA repair genes is a hallmark and a cause of cellular senescence
Source: Cell Death Dis. 2018 Feb 15;9(3):259. doi: 10.1038/s41419-018-0300-z (PMC5833687; doi:10.1038/s41419-018-0300-z)
Supplement: Supplementary file 3 — Supplemental Tables [file 41419_2018_300_MOESM3_ESM.docx]

**Table S1** DNA repair genes down-regulated during OIS in HMEC-MEK cells are regulated by E2F pathway.

|  | | |  |  |
| --- | --- | --- | --- | --- |
|  |  |  |  |  |
| **Gene Name^1^** | **E2Fs ChIP-seq positive^2^** | **FC expression^3^** |  |  |
| **Down-regulated GO DNA repair** | |  |  |  |
| BARD1 | 4 | 0.22 |  |  |
| BLM | 7 | 0.19 |  |  |
| BRCA1 | 5 | 0.17 |  |  |
| BRCA2 | 4 | 0.21 |  |  |
| CDC45 | 5 | 0.19 |  |  |
| CDC7 | 7 | 0.17 |  |  |
| CDCA5 | 6 | 0.18 |  |  |
| CDK1 | 7 | 0.12 |  |  |
| CHAF1A | 5 | 0.19 |  |  |
| DTL | 7 | 0.17 |  |  |
| ESCO2 | 5 | 0.12 |  |  |
| EXO1 | 7 | 0.15 |  |  |
| FANCD2 | 5 | 0.19 |  |  |
| FOXM1 | 5 | 0.23 |  |  |
| HMGB2 | 5 | 0.24 |  |  |
| KIAA0101 | 5 | 0.15 |  |  |
| KIF22 | 5 | 0.24 |  |  |
| LOC100133315 | 2 | 0.21 |  |  |
| NEIL3 | 7 | 0.07 |  |  |
| PARPBP | 5 | 0.10 |  |  |
| PCNA | 7 | 0.20 |  |  |
| PIF1 | 2 | 0.18 |  |  |
| POLE2 | 7 | 0.11 |  |  |
| POLQ | 7 | 0.15 |  |  |
| PTTG1 | 2 | 0.23 |  |  |
| RAD51 | 7 | 0.23 |  |  |
| RAD51AP1 | 4 | 0.08 |  |  |
| RFC3 | 5 | 0.09 |  |  |
| RFC4 | 7 | 0.24 |  |  |
| XRCC3 | 6 | 0.19 |  |  |
|  |  |  |  |  |
|  |  |  |  |  |
| **Up-regulated** |  |  |  |  |
| ACSS3 | 2 | 12.13 |  |  |
| ANK1 | 0 | 35.43 |  |  |
| AQP3 | 0 | 57.42 |  |  |
| ATP8A2 | 0 | 13.72 |  |  |
| CA8 | 0 | 13.68 |  |  |
| CALB1 | 1 | 13.46 |  |  |
| CAMK2B | 0 | 14.50 |  |  |
| CAPN8 | 1 | 12.11 |  |  |
| CAPN8 | 0 | 13.37 |  |  |
| CPO | 0 | 21.72 |  |  |
| DENND2D | 1 | 14.05 |  |  |
| DMBT1 | 2 | 20.86 |  |  |
| FAM43B | 2 | 14.28 |  |  |
| FXYD2 | 0 | 11.64 |  |  |
| GPER1 | 0 | 79.00 |  |  |
| ISYNA1 | 0 | 66.70 |  |  |
| MYH15 | 0 | 55.12 |  |  |
| PLCXD3 | 0 | 15.51 |  |  |
| PRSS35 | 0 | 23.35 |  |  |
| RARRES2 | 0 | 28.67 |  |  |
| SHISA2 | 0 | 11.85 |  |  |
| SLC38A4 | 0 | 40.01 |  |  |
| SLC7A7 | 0 | 23.57 |  |  |
| SNX31 | 0 | 45.97 |  |  |
| SPTSSB | 0 | 165.36 |  |  |
| ST8SIA1 | 0 | 14.45 |  |  |
| SYT12 | 0 | 63.47 |  |  |
| TNFSF15 | 0 | 12.26 |  |  |
| UCA1 | 0 | 24.19 |  |  |
| WIPF1 | 2 | 12.16 |  |  |
|  |  |  |  |  |
|  |  |  |  |  |
| **Down-regulated** |  |  |  |  |
| ACKR3 | 0 | 0.06 |  |  |
| ACTG2 | 0 | 0.03 |  |  |
| AKR1B10 | 0 | 0.05 |  |  |
| AKR1B10 | 0 | 0.06 |  |  |
| BBOX1 | 0 | 0.04 |  |  |
| C1R | 0 | 0.05 |  |  |
| CFB | 0 | 0.06 |  |  |
| CRABP2 | 0 | 0.07 |  |  |
| GBP2 | 0 | 0.05 |  |  |
| GPNMB | 0 | 0.07 |  |  |
| IGFL1 | 0 | 0.06 |  |  |
| LGALS7 | 0 | 0.06 |  |  |
| MMP7 | 0 | 0.03 |  |  |
| MRGPRX3 | 0 | 0.07 |  |  |
| PLIN4 | 0 | 0.08 |  |  |
| RARRES1 | 1 | 0.07 |  |  |
| SAA2 | 0 | 0.05 |  |  |
| SAA4 | 0 | 0.07 |  |  |
| SERPINB13 | 0 | 0.07 |  |  |
| SERPINB3 | 0 | 0.03 |  |  |
| SERPINB4 | 0 | 0.03 |  |  |
| SLC15A1 | 0 | 0.04 |  |  |
| SLC6A14 | 0 | 0.07 |  |  |
| TAGLN | 1 | 0.05 |  |  |
| TENM2 | 0 | 0.06 |  |  |
| TNFAIP6 | 0 | 0.08 |  |  |
| VSNL1 | 0 | 0.07 |  |  |
| VTCN1 | 0 | 0.07 |  |  |
| WFDC2 | 0 | 0.07 |  |  |
| ZNF750 | 0 | 0.05 |  |  |
|  |  |  |  |  |
|  |  |  |  |  |
| **GO DNA repair no down** |  |  |  |  |
| AP5Z1 | 1 | 1.41 |  |  |
| CCNH | 2 | 0.76 |  |  |
| CUL4B | 0 | 2.30 |  |  |
| DDB1 | 1 | 1.00 |  |  |
| EID3 | 0 | 5.66 |  |  |
| ERCC1 | 0 | 3.03 |  |  |
| ERCC5 | 1 | 1.23 |  |  |
| HMGA1 | 4 | 3.73 |  |  |
| HMGA2 | 1 | 1.23 |  |  |
| JMY | 2 | 2.83 |  |  |
| LIG4 | 0 | 2.00 |  |  |
| MC1R | 1 | 2.00 |  |  |
| MNAT1 | 1 | 0.71 |  |  |
| MSH4 | 0 | 1.32 |  |  |
| NABP1 | 1 | 1.74 |  |  |
| NCOA6 | 2 | 1.41 |  |  |
| PARP4 | 2 | 1.74 |  |  |
| POLD4 | 0 | 2.30 |  |  |
| REC8 | 0 | 2.46 |  |  |
| RPA4 | 0 | 3.73 |  |  |
| SETD2 | 0 | 2.00 |  |  |
| SFR1 | 5 | 2.46 |  |  |
| SMG1 | 1 | 1.00 |  |  |
| SPATA22 | 0 | 16.00 |  |  |
| SWI5 | 0 | 0.81 |  |  |
| TRIP12 | 1 | 1.74 |  |  |
| ZFYVE26 | 2 | 2.30 |  |  |
|  |  |  |  |  |
|  | | |  |  |

^1^ The 30 top ranked genes for each class of gene expression (indicated in bold) in response to MEK activation in HMEC cells (HMEC-MEK)

^2^ Number of ChIP-seq experiments showing binding of E2F1 and E2F4 transcription factors for each genes included in the table according to Encode database (Peaks with score above 500).

^3^ Fold Change expression data from microarray experiment we have done in HMEC-MEK cells.

**Table S2** List of qPCR primers.

| **Gene symbol** | **Forward primers** | **Reward primers** | **UPL probe#** |
| --- | --- | --- | --- |
| GAPDH | 5’-agccacatcgctcagacac -3’ | 5’-gcccaatacgaccaaatcc -3’ | 60 |
| BARD1 | 5’-tgaagtgtatgcttgggattctc -3’ | 5’-tcttcgtagacatgcttttaccc -3’ | 89 |
| BLM | 5’-cctcccaaaggtctaagagga -3’ | 5’-gatatctttctacatgtggcagacc -3’ | 38 |
| BRCA1 | 5’-atcattcacccttggcacag -3’ | 5’-catctgcccaattgatgga -3’ | 71 |
| CDKN1A(p21) | 5’-tcactgtcttgtacccttgtgc -3’ | 5’-ggcgtttggagtggtagaaat -3’ | 32 |
| E2F1 | 5’-aagaccccatcccaggag-3’ | 5’-tatggtggcagagtcagtgg-3’ | 63 |
| EXO1 | 5’-cggggttacagatcaaactca -3’ | 5’-tggggacaggggtttctta -3’ | 69 |
| FANCD2 | 5’-caaagctatgctcactctcaaca-3’ | 5’-tcctgggaattttgggactt-3’ | 20 |
| FEN1 | 5’-agaagggagagcgagcttag-3’ | 5’-gggccacatcagcaattagt-3’ | 82 |
| NEIL3 | 5’-aaaaccgaaaacagcccaat -3’ | 5’-cgagggccatctgtcatatt -3’ | 9 |
| POLE2 | 5’-tcctggatttggttccatct-3’ | 5’-ctgtgtacagtactgaattctgcaag-3’ | 49 |
| POLQ | 5’-ctgaaaaatgctgtgcctttc-3’ | 5’-gcgacgttcttcaactgctt-3’ | 8 |
| RAD51 | 5’-atcactaatcaggtggtagctcaa -3’ | 5’-cccctcttcctttcctcaga -3’ | 58 |
| RB | 5’-ggatcagatgaagcagatgga-3’ | 5’-tgcattcgtgttcgagtagaag-3’ | 14 |
| RFC4 | 5’-ccagctgagaaaattgatgga-3’ | 5’-tgctgcatgaccctcatcta-3’ | 27 |
| TP53 | 5’-aggccttggaactcaaggat-3’ | 5’-ccctttttggacttcaggtg-3’ | 12 |
| TRIP13 | 5’-ctcatgcgctgtatgtcca-3’ | 5’-gcttgtccactgccagaga -3’ | 65 |

# Universal ProbeLibrary (UPL) probe number

|  | |  | |  | |  |
| --- | --- | --- | --- | --- | --- | --- |
|  | |  | |  |  |  |
